# Obituary

**Published:** 1887-09

**Authors:** 


### Obituary.

It is with much sorrow that we learn of the death of Louis Cummins, eldest son of Dr. Charles E. and Susanna Dunn, in the tenth year of his age, on Saturday morning, August 20th.

The removal of the young causes a double sadness for all whose sympathies are drawn either to the subject or to the parents. Dr. and Mrs. Dunn will have the warm and heartfelt sympathy of all their friends, and, indeed, of all who know them.

May they have Divine support and comfort in this their great bereavement.

